# High Rate of MCR-1–Producing *Escherichia coli* and *Klebsiella pneumoniae* among Pigs, Portugal

**DOI:** 10.3201/eid2312.170883

**Published:** 2017-12

**Authors:** Nicolas Kieffer, Marta Aires-de-Sousa, Patrice Nordmann, Laurent Poirel

**Affiliations:** Université de Fribourg, Fribourg, Switzerland (N. Kieffer, P. Nordmann, L. Poirel);; Escola Superior de Saúde da Cruz Vermelha Portuguesa, Lisbon, Portugal (M. Aires-de-Sousa);; University of Lausanne and University Hospital Centre, Lausanne, Switzerland (P. Nordmann)

**Keywords:** MCR-1, colistin, pigs, *Escherichia coli*, *Klebsiella pneumonia*, bacteria, Portugal, mcr-1 gene, antimicrobial resistance

## Abstract

The *mcr-1* (mobile colistin resistance 1) gene, which encodes phosphoethanolamine transferase, has been recently identified as a source of acquired resistance to polymyxins in *Escherichia coli*. Using the SuperPolymyxin selective medium, we prospectively screened 100 pigs at 2 farms in Portugal for polymyxin-resistant *Enterobacteriaceae* and recovered 98 plasmid-mediated MCR-1–producing isolates. Most isolates corresponded to nonclonally related *E. coli* belonging to many sequence types; we also found 2 *Klebsiella pneumoniae* sequence types. The *mcr-1* gene was carried on IncHI2 or IncP plasmid backbones. Our finding of a high rate of MCR-1 producers on 2 pig farms in Portugal highlights the diffusion of that colistin-resistance determinant at the farm level. The fact that the pigs received colistin as metaphylaxis in their feed during the 6 weeks before sampling suggests selective pressure.

The progressive global increase of antimicrobial drug resistance in *Enterobacteriaceae* is worrisome, and adding to the concern is the recent discovery of the plasmid-mediated mobile colistin resistance (MCR) genes *mcr-1* and *mcr-2* (*1*,*2*). These genes encode phosphoethanolamine transferases, which add a phosphoethanolamine group to the lipid A of the lipopolysaccharide, leading to gram-negative bacteria resistance to polymyxins (*3*). Since its discovery, the *mcr-1* gene has been identified almost worldwide, mostly in animal and environmental samples (*3*) and to a lesser extent in human clinical samples (*4*). The *mcr-1* gene has often been identified from *Escherichia coli* strains recovered from pigs (*3*,*5*–*8*). More recently, the *mcr-2* gene, which shares 76.8% nt identity with *mcr-1*, has been identified from a single *E. coli* isolate recovered from a pig in Belgium (*2*). The genetic element related to the *mcr-2* gene and possibly involved in its acquisition is insertion sequence (IS) IS*Ec69*.

The *mcr-1* gene has been identified on a large variety of plasmids, such as IncI2, IncX4, IncHI2, IncP, IncFI, IncFII, IncFIB, and IncY (*3*,*9*,*10*). The genetic context of the *mcr-1* gene always includes the *mcr-1* cassette, as previously described (*11*,*12*). In addition, IS*Apl1* is often found upstream of the *mcr-1* gene. It has been recently shown that a second copy of IS*Apl1* may be found downstream of the *mcr-1* gene, therefore bracketing the 2.6-kb *mcr-1* cassette and forming the composite transposon Tn*6330*, demonstrated to be functional and responsible for the transposition of *mcr-1* (*13*,*14*).

We speculate that the emergence and further dissemination of the *mcr-1* and -*2* genes occurred from pigs and that IS*Apl* and IS*Ec69*, respectively, were the main genetic elements involved in that process. We recently demonstrated that *Moraxella* spp. are sources of *mcr*-like encoding genes (*15*); *M. pluranimalium* has been identified as the progenitor of the *mcr-2* gene (*16*). Of note, all *Moraxella* spp. are widespread in pigs (*17*), thus allowing speculation that the whole genetic process that originally led to the emergence of the *mcr-*like genes occurred in those animals.

Worldwide, colistin is widely used in veterinary medicine for different purposes, including treatment of enteric infections, prophylaxis or metaphylaxis (*18*), and as growth promoter in several countries (*19*). Despite this selective pressure, studies reporting identification of colistin-resistant *Enterobacteriaceae* in veterinary medicine remain scarce, although an overall low prevalence of those resistant strains was noticed in Europe (*8*,*10*,*20*,*21*).

To evaluate the prevalence and genetic characterization of colistin-resistant *Enterobacteriaceae* on pig farms, we performed a prospective epidemiologic survey. The study was conducted in Portugal (the fifth largest polymyxin consumer in Europe), where colistin is heavily used in veterinary medicine (*22*).

## Materials and Methods

### Isolates and Susceptibility Testing

On 1 day in June 2016, we collected 100 rectal swab samples from pigs on 2 pig farms in Portugal, 30 km apart. These farms, harboring ≈3,000 pigs each, are production holdings, where piglets are born and fattened before being delivered to slaughterhouses (*23*). All pigs sampled were 10–11 weeks of age. When the pigs were 5–10 weeks of age, their feed included colistin (0.5%), amoxicillin (0.5%), and zinc oxide (0.15%). The weekly dose of colistin in the regimen was ≈0.06 g/kg. Overall, all pigs received ≈5.5 g colistin for metaphylaxis over 6 weeks.

We incubated rectal swab samples overnight at 37°C in Luria-Bertani broth supplemented with 1 μg/mL colistin. The next day, to select for colistin-resistant gram-negative isolates, we inoculated each enrichment tube onto SuperPolymyxin selective agar medium that contained 3.5 μg/mL colistin and 10 μg/mL daptomycin (ELITechGroup, Signes, France) (*24*). We identified colistin-resistant isolates recovered from SuperPolymyxin plates with API 20E (bioMérieux, La Balme les Grottes, France). We performed antimicrobial drug susceptibility testing by using the disk-diffusion method according to Clinical and Laboratory Standards Institute recommendations, on Muller-Hinton agar plates, except for colistin, for which we evaluated MICs for colistin by broth microdilution in cation-adjusted Muller-Hinton broth (Bio-Rad, Cressier, Switzerland), as recommended by the Clinical and Laboratory Standards Institute (*25*).

### Molecular Analyses

Acquired colistin-resistance genes *mcr-*1 and *mcr-*2 were identified by PCR, with use of specific primers as reported (*14*), and amplicons were further sequenced by Microsynth (Balgach, Switzerland). We identified extended-spectrum β-lactamase (ESBL)–encoding genes by using primers specific for detection of *bla*_TEM_, *bla*_SHV_, and *bla*_CTX-M_ genes (*26*,*27*). The plasmidborne chloramphenicol gene *floR* was sought by using specific primers (*28*) among isolates exhibiting resistance to chloramphenicol. The clonal relationship of the colistin-resistant isolates was evaluated by pulsed-field gel electrophoresis, as described previously (*29*), and multilocus sequence typing was performed for a representative strain of each pulsotype. We assigned sequence types (STs) by using the multilocus sequence typing databases for *E. coli* (http://mlst.warwick.ac.uk/mlst/dbs/Ecoli) and *K. pneumoniae* (http://bigsdb.pasteur.fr/klebsiella/klebsiella.html). The phylogenetic group of *E. coli* isolates was determined with the PCR-based Clermont method as described previously (*30*).

### Conjugation Experiments and Plasmid Analyses

We performed conjugation assays on filters with azide-resistant *E. coli* J53 as the recipient strain. MCR-1 producers and the J53 isolate were cultured overnight in Luria-Bertani broth. To reach the logarithmic phase, the donor and recipient strains were subcultured in fresh Luria-Bertani broth for 3 h. We subsequently mixed the samples at a ratio of 10:1 and deposited 100 μL of this mix onto 22-μm filters, which we then incubated for 5 h at 37°C on Luria-Bertani agar plates. After the incubation, filters were resuspended in 0.85% NaCl, and we plated 250 μL of this mixture onto selective Luria-Bertani plates containing azide (100 μg/mL) and colistin (1 μg/mL). PCR was used to check all *E. coli* transconjugants for the *mcr-1* gene.

We typed plasmids carrying the *mcr-1* gene from *E. coli* transconjugants by using the PCR-based replicon typing method (*31*). The size of the plasmid was obtained after Kieser extraction (*32*) and agarose gel electrophoresis; we used as a reference *E. coli* 50192 isolate containing 4 characterized plasmids (154 kb, 66 kb, 48 kb, and 7 kb).

## Results

From the 100 rectal swab samples collected, we recovered 108 colistin-resistant isolates from the SuperPolymyxin agar plates and identified 90 as *E. coli*, 17 as *K. pneumoniae*, and 1 as *Proteus mirabilis.* Of the 108 colistin-resistant isolates, 98 were positive for the *mcr-1* gene. Colistin MICs for all MCR-1–producing isolates ranged from 4 to 32 μg/mL (Table). Among these positive isolates, 10 showed an ESBL phenotype. Sequencing revealed that all *mcr*-positive isolates possessed a gene that was 100% identical to *mcr-1.* All MCR-1–producing isolates possessed the *bla*_TEM-1_ gene, and all ESBL producers possessed the *bla*_CTX-M-2_ gene. All MCR-1–producing isolates were resistant to penicillins and tetracycline, 97.9% were resistant to sulfamethoxazole/trimethoprim, 96% were resistant to tobramycin, and 84.7% were resistant to chloramphenicol. Among the chloramphenicol-resistant isolates, 56% were positive for the *floR* resistance gene. No *mcr-2*–positive isolate was identified in our samples.

**Table:** Genetic features associated with MCR-1–producing *Escherichia coli* and *Klebsiella pneumoniae* isolates from pigs, Portugal*

Strain	Species	No. pigs	ST†	Resistance genes	Colistin MIC, μg/mL	Incompatibility group (kb) of *mcr-1* plasmids	Resistance phenotype‡	Genetic context of *mcr-1*§
Farm 1								
P13	*E. coli*	6	ST101	*bla*_CTX-M-2_, *bla*_TEM-1_, *mcr-1*	4	IncHI2 (≈250)	SXT/TET/NAL/AMX/CTX/TMN/SUL	II
P8	*E. coli*	1	ST101	*bla*_CTX-M-2_, *bla*_TEM-1_, *mcr-1, floR*	4	IncHI2 (≈250)	SXT/TET/AMX/CTX/TMN/SUL	II
P28	*E. coli*	4	New ST_1_	*bla*_TEM-1_, *mcr-1, floR*	8	IncHI2 (≈250)	SXT/TET/CHL/AMX/TMN/GMN/SUL	II
P11	*E. coli*	1	New ST_2_	*bla*_TEM-1_, *mcr-1, floR*	16	IncHI2 (≈250)	SXT/TET/CHL/AMX/TMN/SUL	III
P4	*E. coli*	3	New ST_1_	*bla*_TEM-1_, *mcr-1*	16	IncP (≈60)	TET/ CHL /AMX/TMN/SUL	II
P9	*E. coli*	1	New ST_2_	*bla*_TEM-1_, *mcr-1*	16	IncP (≈60)	TET/CHL/AMX/TMN/SUL	II
P27	*E. coli*	1	ST6453	*bla*_TEM-1_, *mcr-1*	16	IncP (≈60)	TET/CHL/AMX/TMN/SUL	I
P7	*E. coli*	1	New ST_2_	*bla*_TEM-1_, *mcr-1*	16	IncP (≈60)	TET/CHL/AMX/TMN/SUL	II
P43	*E. coli*	1	New ST_4_	*bla*_TEM-1_, *mcr-1*	16	IncP (≈60)	TET/CHL/AMX/TMN//GMN	III
P10	*E. coli*	1	ST10	*bla*_TEM-1_, *mcr-1*	16	IncHI2 (≈250)	TET/ CHL /AMX/TMN/SUL	II
P5	*E. coli*	1	New ST_4_	*bla*_TEM-1_, *mcr-1*	8	IncP (≈60)	TET/ CHL /AMX/TMN/SUL	II
P1	*E. coli*	1	New ST_3_	*bla*_TEM-1_, *mcr-1*	8	IncP (≈60)	TET/ CHL /AMX/TMN/SUL	II
P2	*E. coli*	1	New ST_2_	*bla*_TEM-1_, *mcr-1*	8	IncP (≈60)	SXT/TET/CHL/AMX/TMN/SUL	II
P16	*E. coli*	1	ST10	*bla*_TEM-1_, *mcr-1*	8	IncX4(≈30)	SXT/TET/AMX/TMN/SUL	III
P3	*E. coli*	5	ST156	*bla*_TEM-1_, *mcr-1*	8	IncHI2 (≈250)	SXT/TET/NAL/CIP/AMX/TMN/SUL	I
P20	*E. coli*	1	New ST_2_	*bla*_TEM-1_, *mcr-1*	4	IncHI2 (~250)	SXT/TET/AMX/TMN/SUL	III
P22	*E. coli*	13	ST6453	*bla*_TEM-1_, *mcr-1*	4	IncP (≈60)	TET/AMX/TMN/SUL	I
P19	*E. coli*	1	New ST_4_	*bla*_TEM-1_, *mcr-1*	4	IncP (≈60)	SXT/TET/ CHL/NAL/AMX/TMN/SUL	II
P37	*E. coli*	1	ST38	*bla*_TEM-1_, *mcr-1*	4	IncP (≈60)	SXT/TET/AMX/TMN/SUL	I
P6K	*K. pneumoniae*	6	ST45	*bla*_TEM-1_, *mcr-1*	32	IncP (≈60)	TET/AMX/TMN/SUL	II
Farm 2								
B21	*E. coli*	1	ST10	*bla*_TEM-1_, *mcr-1, floR*	16	IncHI2 (≈250)	SXT/CHL/TET/NAL/CIP/AMX/TMN/SUL	III
B12	*E. coli*	8	ST10	*bla*_TEM-1_, *mcr-1, floR*	16	IncHI2 (≈250)	SXT/CHL/TET/NAL/CIP/AMX/TMN/SUL	III
B30	*E. coli*	10	ST10	*bla*_TEM-1_, *mcr-1, floR*	16	IncHI2 (≈250)	SXT/CHL/TET/NAL/CIP/AMX/TMN/SUL	III
B3	*E. coli*	1	New ST_6_	*bla*_TEM-1_, *mcr-1, floR*	16	IncHI2 (≈250)	SXT/CHL/TET/NAL/AMX/TMN/SUL	I
B27	*E. coli*	1	New ST_7_	*bla*_TEM-1_, *mcr-1, floR*	16	IncHI2 (≈250)	CHL/TET/NAL/CIP/AMX/TMN/SUL	I
B47	*E. coli*	1	New ST_7_	*bla*_TEM-1_, *mcr-1, floR*	16	IncHI2 (≈250)	SXT/CHL/TET/AMX/TMN/SUL	III
B18	*E. coli*	2	New ST_8_	*bla*_TEM-1_, *mcr-1, floR*	16	IncX4 (≈30)	SXT/CHL/TET/AMX/TMN/SUL	III
B22	*E. coli*	5	New ST_8_	*bla*_TEM-1_, *mcr-1, floR*	16	IncHI2 (≈250)	SXT/CHL/TET/AMX/TMN/SUL	II
B4	*E. coli*	1	New ST_7_	*bla*_TEM-1_, *mcr-1, floR*	16	IncHI2 (≈250)	SXT/CHL/TET/AMX/TMN/SUL	III
B15	*E. coli*	1	ST46	*bla*_TEM-1_, *mcr-1, floR*	8	IncHI2 (≈250)	SXT/CHL/TET/AMX/TMN/SUL	III
B6	*E. coli*	2	ST101	*bla*_TEM-1_, *mcr-1, floR*	8	IncHI2 (≈250)	SXT/CHL/TET/AMX/TMN/SUL	III
B8	*E. coli*	1	New ST_6_	*bla*_TEM-1_, *mcr-1, floR*	8	IncX4 (≈30)	SXT/CHL/TET/NAL/AMX/TMN/SUL	III
B1	*E. coli*	1	New ST_6_	*bla*_TEM-1_, *mcr-1, floR*	8	IncHI2 (≈250)	SXT/CHL/TET/AMX/TMN/SUL	III
B11	*E. coli*	1	New ST_7_	*bla*_TEM-1_, *mcr-1, floR*	8	IncHI2 (≈250)	CHL//TET/NAL/AMX/TMN/SUL	IV
B9	*E. coli*	9	ST23	*bla*_TEM-1_, *mcr-1, floR*	4	IncP (≈60)	SXT/CHL/TET/NAL/AMX/TMN/SUL	II
B5	*E. coli*	1	New ST_8_	*bla*_TEM-1_, *mcr-1, floR*	4	IncP (≈60)	SXT/CHL/TET/NAL/AMX/TMN/SUL	III
B27K	*K. pneumoniae*	2	ST1563	*bla*_TEM-1_, *mcr-1, floR*	32	IncHI2 (≈250)	SXT/CHL/TET/AMX/TMN/SUL	I

*Only strains representative of each pulsed-field gel electrophoresis clonal lineage are listed. AMX, amoxicillin; CIP, ciprofloxacin, CHL, chloramphenicol; CTX, cefotaxime; NAL, nalidixic acid; ND, not determined; ST, sequence type; SUL, sulfonamides; SXT, sulfamethoxazole/trimethoprim; TET, tetracycline; TMN, tobramycin. †STs were identified with the Warwick University database (http://mlst.warwick.ac.uk/mlst/dbs/Ecoli). Because this database accepts only whole-genome sequencing data to submit new STs, all new STs found were notified as New ST_x_. ‡Underlining indicates co-resistances provided by the plasmid carrying *mcr-1*. §Different genetic environments detected by PCR, as shown in the Figure.

Pulsed-field gel electrophoresis identified 19 distinct *E. coli* clones from the first farm sampled and 18 from the second (Table). The isolates belonged to 15 STs (ST10, ST23, ST38, ST46, ST101, ST156, ST6453, and 8 new STs); only 2 (ST10 and ST101) were detected on both farms. Phylogenetic typing showed that each *E. coli* isolate belonged to 1 of the phylogroups A, B1, C, D, E, or F. No extraintestinal and virulent B2 phylogroup was detected among all *E. coli* isolates. In addition, we identified 2 *K. pneumoniae* STs (1 clone per farm), ST45 and ST1563.

Conjugation followed by PCR-based replicon typing analysis showed that the *mcr-1* gene was carried on different plasmids (Table). The *mcr-1* gene was identified on IncHI2 (54%), IncP (38%), and IncX4 (8%) plasmids. Conjugation experiments showed that resistance to sulfamethoxazole/trimethoprim and sulfonamides was systematically co-transferred along with the *mcr-1* gene when carried by IncHI2 plasmids and that resistance to tetracycline, tobramycin, chloramphenicol, and amoxicillin was also most often co-transferred by IncP plasmids (Table). Conversely, the *mcr-1* gene was the only resistance determinant when located on IncX4 plasmids. Of note, IncP-type plasmids carrying *mcr-1* were predominant on the first farm, whereas IncHI2 plasmids were predominant on the second farm.

By PCR mapping using previously published primers (*12*,*13*), in all isolates we identified the ≈2.6-kb *mcr-1* cassette in association with 0, 1, or 2 copies of insertion sequence IS*Apl1*, depending on the isolates tested (Table; Figure). In addition, we found a genetic structure not previously reported in 1 isolate that consisted of a truncated IS*Apl1* element upstream of the *mcr-1* cassette. That structure, inserted into a kinase gene onto an IncHI2 plasmid, corresponded to a truncated version of transposon Tn*6330* previously reported, with a 2-bp AG direct repeat bracketing the ∆IS*Apl1*-*mcr-1* structure, suggesting a former insertion event through a transposition mechanism (Figure).

**Figure:**
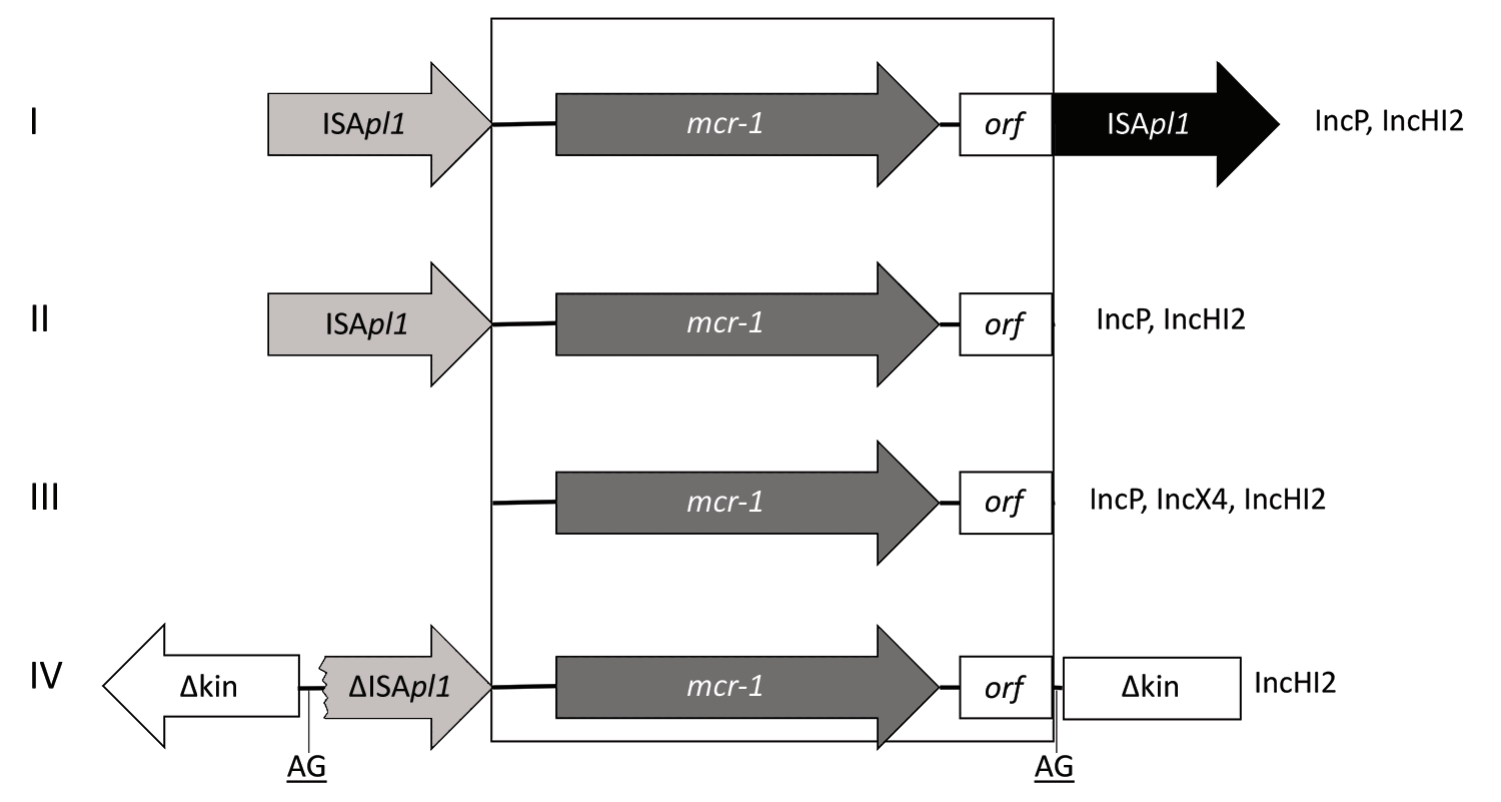
Genetic environments associated with the *mcr-1* (mobile colistin resistance 1) gene detected in select *Enterobacteriacae* isolates from pigs, Portugal, by PCR. I) 1 copy of IS*Apl1* associated with *mcr-1* in 5′ region; II) 2 copies of IS*Apl1* in 5′ and 3′ regions of *mcr-1*; III) no copy of IS*Apl1* associated with *mcr-1*; IV) truncated copy of IS*Apl1* associated with *mcr-1* in 5′ region inserted in a kinase gene. IS, insertion sequence; *orf*, open reading frame.

## Discussion

Prevalence of MCR-1–producing *Enterobacteriacae* isolates, mainly *E. coli*, from 2 pig farms in Portugal was high. So far, the *mcr-1* gene has been identified mainly in animal samples, rarely in environmental and human samples (*3*,*4*,*33*–*36*). Previous studies describe the occurrence of MCR-1 producers in swine; prevalence in Europe ranged from 0.5% to 13.5% (*6*,*8*,*36*,*37*) and in China up to 20.6% (*1*). In our study, we detected an extremely high rate of MCR-1–producing *Enterobacteriaceae*, finding the *mcr*-1 gene in isolates from 98% of pigs tested. We identified an MCR-1–producing *K. pneumoniae* among the pigs, which is noteworthy considering the infrequent recovery of *K. pneumoniae* from animals. Nevertheless, most of the *mcr-1*–positive isolates identified here were *E. coli*, as is reported in most epidemiologic studies (*3*). For the same pigs that had been screened (by nasal swabs) for methicillin-resistant *Staphylococcus aureus*, the rate of colonization was very high (99%); 2 main *spa* types of clone ST398 were identified (*23*).

Our study was performed with samples from pigs, and it would be of interest to conduct similar studies of humans. In France, a survey performed in a hospital during February–May 2016 (*38*) showed a high rate (23%) of fecal carriage with intrinsic colistin-resistant gram-negative isolates but a low rate (1.4%) of acquired polymyxin resistance; no *mcr-1* or *mcr-2* genes were identified. A retrospective study focusing on *Salmonella* isolates was previously performed in Portugal, and MCR-1 producers were reportedly found in humans and pork (*39*). No MCR-2–producing isolate was identified in our study, although this gene was also identified in pigs (*2*).

We used the newly developed SuperPolymyxin medium for our prospective epidemiologic study. The fact that no colistin-susceptible strain was recovered during the screening further highlights the excellent specificity of this medium. 

Unexpectedly, we found that the studied collection of MCR-1 producers was highly diverse; we identified many STs and genetic features associated with *mcr-1*. The rate of *mcr-1* in our study was very high and caused by the dissemination of neither a single clone nor a plasmid. This high diversity could be explained by 2 key elements. First, considering that the selective pre-enrichment with Luria-Bertani broth supplemented with 1 μg/mL of colistin and subsequent culturing on the Superpolymyxin medium exhibits a high sensitivity, such a prospective survey may detect higher rates of colistin-resistant isolates than would previous studies. Second, the presence of colistin in the pig food on the 2 farms studied probably represents an efficient selective pressure for MCR-1 acquisition. Findings of similar and comparative studies performed in countries that do not use polymyxins in animals, such as Norway or Finland, would be of interest.

Several STs of *E. coli* identified in this study were similar to those of other MCR-1– producing isolates reported from other studies. As an example, ST10 *E. coli* producing MCR-1 was recovered from swine farms in Germany and in clinical samples in India and South Africa (*6*,*11*,*40*). In South Africa, ST101 *E. coli* was identified from a patient with a urinary tract infection (*11*). ST156 *E. coli* was identified at a hospital in China and in a muscovy duck in China, where it was co-producing MCR-1 and NDM (New Delhi metallo-β-lactamase)–5 carbapenemase (*41*,*42*). We showed that all *E. coli* isolates belonged to a commensal population and not to extraintestinal pathogenic strains, which is consistent with findings of other studies (*3*) and in line with the design of our study, which was analyzing the colonizing gut flora.

We showed that the *mcr-1* gene was carried by a diversity of plasmids. However, most plasmids recovered from the first farm were ≈60-kb IncP plasmids, whereas those from the second farm were ≈250-kb IncHI2 plasmids. Only 3 STs possessed a ≈30-kb IncX4 plasmid carrying the *mcr-1* gene, which contrasts with other studies that have shown this type of plasmid to be predominant (*43*). In accordance with what has been observed with other characterized IncX4 plasmids, we found no other resistant determinant associated with *mcr-1* on that plasmid type. IncP and IncHI2 plasmids carried other resistance determinants associated with *mcr-1*. Remarkably, we found no IncI2-type plasmid carrying *mcr-1* in those isolates, although they have often been reported in the literature (*1*,*3*).

Analysis of the genetic features associated with the *mcr-1* gene further highlights that it was probably originally acquired by a transposition mechanism and that IS*Apl1* played a major role; further truncations or rearrangements led to the stabilization of this structure, as suggested by Snesrud et al. (*44*). We also identified the entire composite transposon Tn*6330* comprising 2 copies of IS*Apl1* bracketing the *mcr-1* cassette (*13*). Because this entire transposon was detected in some isolates of this collection in addition to other defective versions of it, we can speculate that this structure may still be mobilizable and continue to disseminate between different genetic locations.

In summary, the rate of pig colonization with MCR-1–producing *Enterobacteriaceae* was high at the 2 farms we sampled, showing substantial diversity of species, clonality, and genetic aspects. Even if these results suggest that colistin constitutes a major driving force for selecting plasmids carrying the *mcr-1* gene, the occurrence of the *bla*_TEM-1_ gene on the same plasmid indicates that β-lactams might also be co-selecting for colistin resistance through the acquisition of such plasmids. In addition, this study showed that SuperPolymyxin is an efficient medium for screening colistin-resistant isolates from animal samples and performing such epidemiologic surveys. Last, considering that a recent report from Germany identified pig farms as potential sources of environmental contamination for MCR-1–producing *E. coli* (*6*), our data strongly indicate the need for screening farm environments in Portugal, to evaluate the extent to which the spread of those resistant bacteria has already occurred and, therefore, to better measure the risk to human health.
